# AI-based approach for heart failure readmission prediction using SCG, ECG, and GSR signals

**DOI:** 10.1088/1361-6579/ae178c

**Published:** 2025-11-04

**Authors:** Rajkumar Dhar, Md Rakib Hossen, Peshala T Gamage, Richard H Sandler, Nirav Y Raval, Robert J Mentz, Hansen A Mansy

**Affiliations:** 1 Quantitative Health Science, Lerner Research Institute, Cleveland Clinic, Cleveland, OH 44195, United States of America; 2 Biomedical Acoustic Research Lab, University of Central Florida, Orlando, FL 32816, United States of America; 3 Florida Tech, Melbourne, FL 32901, United States of America; 4 Biomedical Acoustics Research Company, Orlando, FL, United States of America; 5 Advent Health, Orlando, FL, United States of America; 6 Duke University, Durham, NC 27715, United States of America

**Keywords:** seismocardiogram, heart failure readmission, signal processing, machine learning

## Abstract

*Objective.* Heart failure (HF) is considered a global pandemic because of increasing prevalence, high mortality rate, frequent hospitalization, and associated economic burden. This study explores a noninvasive method that may help in managing HF patients by predicting HF readmission. *Methods.* Seismocardiogram (SCG) signal is the low-frequency chest vibration produced by the mechanical activity of the heart. SCG signal was acquired from 101 patients with HF, including those readmitted to the hospital during the study period. SCG signals were segmented into heartbeats and clustered based on respiration phases. Features were extracted from each cluster. Several conventional machine learning (ML) models were developed using selected SCG and heart rate variability features. Furthermore, SCG signals were transformed into images using a time–frequency distribution method. Images were used to train a deep learning model. The models were able to predict the readmission status of HF patients. *Results.* ML algorithms achieved higher accuracy than the deep learning model in classifying the readmitted and non-readmitted HF patients. K-nearest neighbor achieved the highest classification accuracy (89.4% accuracy, 87.8% sensitivity, 90.1% specificity, 78.2% precision, and 82.7% *F*1-score). A detailed discussion of the extracted features was provided, correlating them with HF conditions. *Conclusions*. The study results suggest that SCG signals may be useful for readmission prediction of HF patients.

## 1. Introduction

Heart failure (HF) is a chronic progressive medical condition marked by the diminished capacity of the heart to effectively pump blood. HF is a major global health concern with an estimated 64 million cases worldwide (Savarese and Lund 2017) and 6 million in the United States (Virani *et al* 2020). This is projected to rise to 8.5 million in 2030 in the US (Bozkurt *et al* 2023). This increasing prevalence mainly accounts for the aging populations who are at greater risk of developing HF. Advances in medical diagnosis and treatment have improved survival rates, prolonging life in individuals with HF (Savarese and Lund 2017). Nevertheless, the mortality rate related to HF is still very high. A meta-analysis by Jones *et al* in 2018 showed that the 1- and 5 year survival rates of HF are 86.5% and 56.7%, respectively (Jones *et al* 2019). According to a more recent study by Bozkurt *et al*, 28% of 263 525 patients died during the first year of first HF hospitalization (2023). Apart from this, the healthcare costs related to HF is also substantial (Lesyuk *et al* 2018). The total cost for HF was estimated at $43.6 billion in the US, which is projected to increase to $70 billion by 2030 (Urbich *et al* 2020, Heidenreich *et al* 2022). The main driver of HF healthcare cost is hospitalization (Shafie *et al* 2018), as HF is associated with a very high number of hospital readmission rates. After discharge, about 25% and 50% of HF patients are readmitted within the 30 d and 6 month periods, respectively (Virani *et al* 2020, Khan *et al* 2021). With the increase in HF prevalence, the readmission rate and associated costs are likely to be increased in the coming years. Therefore, early readmission prediction may allow interventions that may reverse patient deterioration and avoid readmission.

HF can be classified based on left ventricular ejection fraction (LVEF). LVEF is the fraction of blood pumped out of the heart’s left ventricle (LV) during systole. It provides a measurement of LV systolic function, which is responsible for ejecting oxygenated blood from the heart to the rest of the body. Normal range of LVEF is 50%–70% (Lang *et al* 2015). Classification of HF regarding LVEF is illustrated in table 1.

**Table 1.** Classification of HF according to LVEF. Here, HFrEF is HF with reduced ejection fraction, HFmEF is HF with mildly reduced ejection fraction, and HFpEF is HF with preserved ejection fraction (Heidenreich *et al* 2022). LVEF stands for left ventricular ejection fraction.

HF class	LVEF
HFrEF	⩽40%
HFmrEF	41%–49%
HFpEF	⩾50%

HFrEF comprises approximately 50% of total HF cases (Murphy *et al* 2020). Patients with HFrEF have a higher mortality rate than those with HFpEF (Somaratne *et al* 2009, Burkhoff 2012). Although all-cause readmission is higher in HFpEF, HF readmission is higher in HFrEF (Cui *et al* 2020). In addition, the cost of readmission is higher in HFrEF patients (Sheikh *et al* 2021). Regardless of the HF class, the high readmission rate is avoidable with preventive measures (Desai and Stevenson 2012). In -(Stauffer 2011), it was demonstrated that a post-discharge transitional care program can greatly reduce the HF readmission rate and the associated cost. Taking this into account, continuous efforts have been made to build an early and reliable HF readmission prediction model that may help the clinicians to make timely targeted interventions to prevent readmissions.

Electronic health records (EHRs) and wearable sensors are the main data sources that have been used to predict HF readmission. EHR includes patient demographics, medications, vital signs, medical history, laboratory data, etc. Intrathoracic impedance, electrocardiogram (ECG), and seismocardiogram (SCG) can be acquired with wearable devices and used as predictors of HF readmission. The predictive accuracy values of these studies are widely varied. In Shameer *et al* (2016), authors used EHR data and achieved 83.19% accuracy in 1068 patients. In another study, sensitivity and specificity of 48% and 70% are achieved, respectively, using medical data of 10 757 HF patients (Awan *et al* 2019). A review article by Liu *et al* showed that B-type natriuretic peptide (BNP) and N-terminal pro-brain natriuretic peptide (NT-proBNP) are the most used predictors from the EHR data (Liu *et al* 2022).

Other authors used sensor data to predict HF readmission. Intrathoracic impedance-based models obtained variable predictive accuracy ranging from 21%–76%, suggesting the uncertainty in predicting HF readmission (Yu *et al* 2005, Cleland and Antony 2011, Heist *et al* 2014, Stehlik *et al* 2020). In (Stehlik *et al* 2020), ECG, skin impedance, temperature, etc were acquired from 100 patients at home with a multisensory patch for 3 months. High prediction accuracy was achieved (sensitivity = 86%, specificity = 87.5%) using the sensor data, although the study required baseline data for analysis. Boehmer *et al* used defibrillators implanted in patients to acquire data to predict hospitalization (Boehmer *et al* 2017). Invasive accelerometer-acquired heart sounds (similar to SCG), heart rate, intrathoracic impedance, respiration rate, and tidal volume data were collected from the implanted device, which were able to alert clinicians before HF hospitalization (sensitivity = 70%). In another SCG-based study, Lin *et al* identified HF patients by calculating LVEF from SCG and ECG signals (Lin *et al* 2018). In the study, 40 subjects were enrolled (25 HF and 15 healthy). The ratio of pre-ejection period and left ventricular ejection time was calculated from SCG and ECG signals, which was found to be inversely proportional to LVEF (correlation coefficient 0.73). A threshold ratio of 0.33 distinguished HF from healthy participants with 96% accuracy (sensitivity 98% and specificity 94%). Inan *et al* used SCG signals to distinguish between compensated and decompensated HF patients (2018). The patients needed to perform the 6 min walk test (6MWT) in this study. Similarity between SCG signals before and after the test was used as a metric to differentiate the two groups. Higher similarity was found in decompensated patients, suggesting their reduced cardiovascular reserve. Although the above studies had several limitations, such as requiring baseline data, demanding patients to perform 6MWT, or using invasive measurements, these studies demonstrated the merit of SCG signal in predicting HF readmission. The current study investigates the feasibility of using SCG and machine learning (ML) algorithms for HF readmission prediction when baseline measurements are not available.

## 2. Materials and method

### 2.1. Data acquisition

The dataset used in this study was collected at Advent Health Orlando after IRB approval by the University of Central Florida (protocol number: BIO-16-12783; the date of approval: March 6, 2023). The study was carried out according to the principles outlined in the Declaration of Helsinki. HF patients were recruited after their discharge from the hospital. Overall, 101 patients were included in this study. Data was acquired in single or multiple sessions per patient, following their provision of written informed consent. After an observer manually checked the data, 24 recording sessions were excluded due to poor quality of the acquired signals (zero voltage or noisy signal). This resulted in the exclusion of 20 patients from the study. Data analysis was performed in the remaining 81 patients who had a total of 142 sessions. The demographic information of the subjects is shown in table 2.

**Table 2.** Available demographics. Age information was not available.

Category	Details
Gender	Male: 62, female: 19
Height (m)	1.74 ± 0.11
Weight (kg)	101.7 ± 30.7
BMI (kg m^−2^)	33.3 ± 9.4
HF status	HFrEF: 75, HFpEF: 6
NYHA classification^a^	I: 2, II: 14, III: 28, IV: 19

^a^ NYHA classification was reported in 63 subjects.

After the initial discharge, 22 patients (who attended 41 recording sessions) were readmitted to the hospital during the window of data acquisition (six months). The protocol included 3 min of data acquisition in each session when patients were sitting on a 45° inclined exam table with their legs extended. The following three signals were acquired from the patients:

i. SCG: Acquired using a tri-axial accelerometer (Model: 356A32, PCB Piezotronics, Depew, NY) placed on the chest surface at the 4th intercostal space near the left lower sternal border. Signal was amplified using a signal conditioner (Model: 482C, PCB Piezotronics, Depew, NY) with a gain of 100. The *x, y*, and *z* components of the accelerometer are pointed toward lateral (left to right), caudocranial (head to toe), and dorsal–ventral (normal to chest surface) directions, respectively. This study includes the analysis of the *z*-axis of the accelerometer.
ii. ECG): Acquired by IX-B3G bio-potential recorder (iWorx Systems, Inc., Dover, NH).
iii. Galvanic skin response (GSR): Provides an estimate of lung volume (Azad *et al* 2018). Acquired by IX-B3G bio-potential recorder.

**Figure 1.**
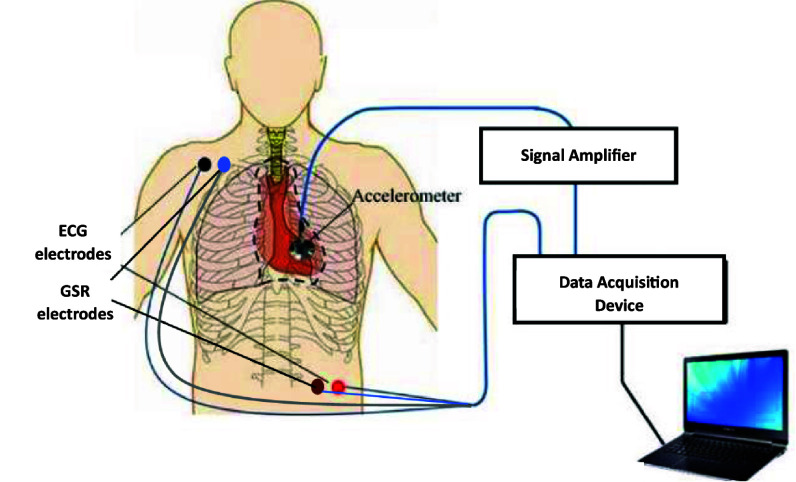
Schematic of experiment setup.

All the signals were acquired at a sampling rate of 10 kHz. A schematic representation of data acquisition is shown in figure 1.

### 2.2. Data analysis

**Figure 2.**
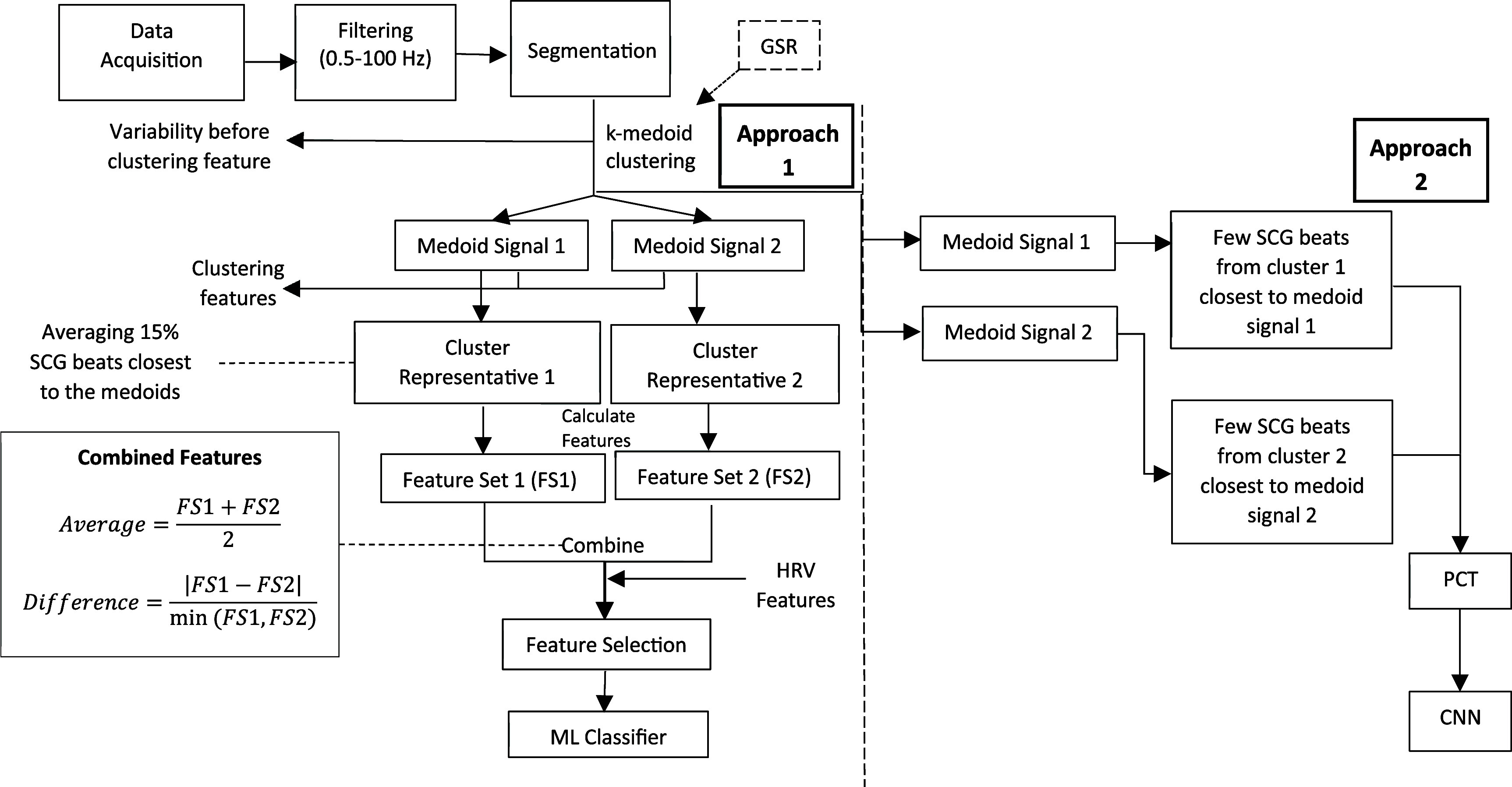
Flow diagram of data analysis.

Overview: The workflow diagram of data analysis is shown in figure 2. The process started with filtering raw signals (band pass = 0.5–100 Hz), followed by the segmentation of SCG and ECG signals (Azad *et al* 2023). After that, SCG beats were clustered using an unsupervised clustering method (k-medoids clustering) (Gamage *et al* 2020). The clustering was correlated to the respiration phases, which were obtained from GSR signal. This clustering provides a medoid SCG beat for each cluster. Clustering features (described below) were extracted using the relationship between the medoid SCG beats and the rest of the SCG beats. Other time- and frequency-domain features were extracted from the cluster ‘representative’ beats (described below). Conventional ML models were trained and tested using selected SCG features along with a few heart rate variability (HRV) features. This concludes the first approach of analysis that utilizes conventional ML.

In the second approach, a few SCG beats (3–5) that were closest (in terms of waveform shape) to the medoid beats were transformed into images using a time–frequency distribution method (polynomial chirplet transform or PCT). The images were fed to a CNN model for training and testing.

#### 2.2.1. Preprocessing

After visually checking the signal quality, noisy portions of the data were discarded. This noise mainly came from patient movements. The rest of the data (usually 100–140 s) was considered for analysis. The raw ECG, SCG, and GSR signals were downsampled to 1 kHz. After that, ECG and SCG signals were forward–backward filtered using a 4th order Chebyshev type 2 bandpass filter with cutoff frequencies of 0.5 and 100 Hz. The GSR signal was detrended, and a flow rate signal was calculated by differentiating the GSR signal.

#### 2.2.2. Segmentation and normalization

The R-peaks of the ECG signal were detected using the Pan–Tompkins algorithm (Tompkins 1985). SCG and ECG beats were chosen to start 0.1 s before the ECG R-wave and end 0.1 s before the next R-wave. After segmentation, each SCG beat was normalized by its peak-to-peak amplitude.

#### 2.2.3. Unsupervised clustering (k-medoid clustering)

**Figure 3.**
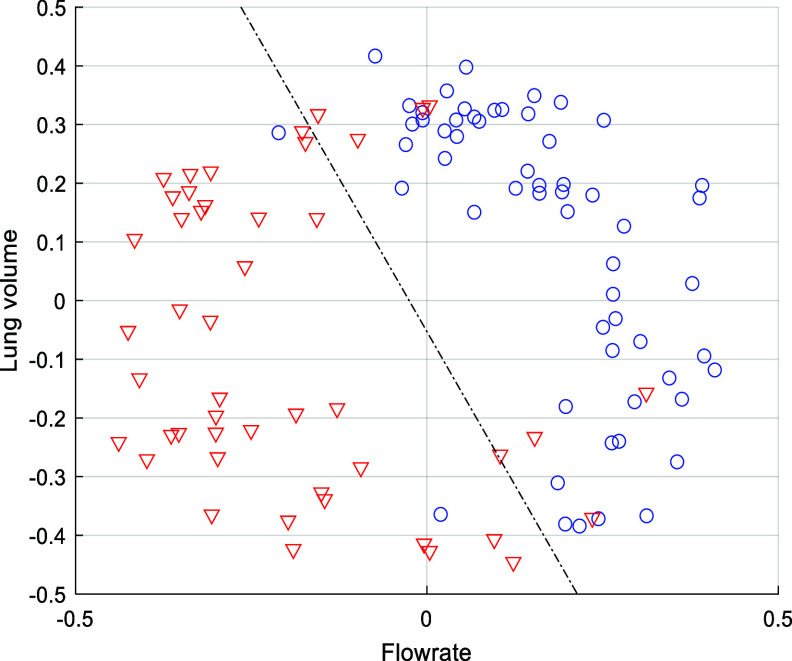
K-medoid clustering of SCG beats of a representative recording session (Subject 25, 3rd session) in lung volume-flow rate space. Blue circles and red triangles are the beats of the two clusters. A decision boundary (dashed line) is plotted to show the clear separation between the two clusters.

Studies on SCG signals reported that SCG signals have morphological variability (Azad *et al* 2019, Sandler *et al* 2019, Gamage *et al* 2020). The clusters of similar SCG beats were found to correlate with the respiration phases. It was suggested that clustering SCG beats into two clusters optimally lowers the variability and makes the feature extraction more accurate (Gamage *et al* 2020). To group the SCG beats with close morphological features, the k-medoids clustering method was used. The unsupervised clustering method requires two initial beats. Efficient clustering depends on good initialization. In the current study, the SCG beats are initially divided into two groups based on either lung volume (high and low) or flow rate (high and low). SCG beats are considered to be more similar when the distance between them is smaller. Dynamic time warping (DTW) and cross-correlation methods are the two methods chosen to measure the distance (i.e. morphological dissimilarity) between the SCG beats. After dividing the beats into two groups based on lung volume and flow rate, center beats were chosen from each group that had the minimum sum of distances with their neighboring beats in the same group. These two center beats are chosen as the initial beats for the k-medoids method, which is named as initial medoids. After obtaining the initial medoids, the clustering process began. The algorithm continued to update the cluster medoids by calculating the sum of distances and then update the clusters by grouping the beats that have morphological similarities measured by DTW distance. The algorithm stopped when there was no change in the assignment of the SCG beats to the clusters in two consecutive iterations. As there were two bases of grouping (lung volume and flow rate) and two distance measuring methods (DTW and cross-correlation), all four combinations of getting the initial medoids were performed. The combination that produced the most optimum clustering of SCG beats was selected. Clustering quality was also checked by plotting the clustered beats in a lung volume-flow rate space (figure 3). A decision boundary was drawn to visualize the separation of the beats into two clusters.

After getting the cluster medoids, 15% of SCG beats that are closest (measured by DTW distance) to the medoid signal in a cluster were averaged to create a SCG beat that is a representative of that cluster. Features were extracted from both cluster medoids and cluster representatives.

#### 2.2.4. Feature extraction and selection

**Figure 4.**
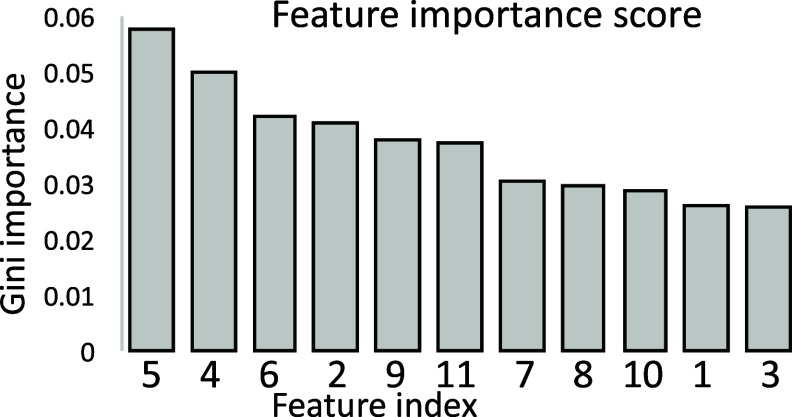
Feature importance scores of input variables computed using random forest based on Gini importance.

In total, 63 SCG features were extracted. These include clustering, time- and frequency-domain features. In addition, 8 HRV features were added to complete the feature set. The random forest (RF) algorithm was employed for feature selection. RF is a popular and powerful algorithm that falls under the embedded feature selection method. This embedded method combines the benefits of the other two feature selection methods (filter and wrapper) by allowing interaction with the classifier (like the wrapper method) and being computationally lighter while at the same time producing better classification results (Guo *et al* 2019, Pudjihartono *et al* 2022). 11 features were selected (7 SCG and 4 HRV features). A list of selected features is given in table 3, and the feature importance scores are provided in figure 4.

**Table 3.** Selected SCG (1–7) and HRV (8–11) features with short descriptions. The features (4–7) are obtained by averaging the features from the two cluster representative waveforms (for each recording session).

Feature index	Feature name	Description	
1	Intra-session waveform variability before clustering (WV_bc_).	The dissimilarity among the SCG beats within a session. Dissimilarity was calculated using dynamic time warping (*dtw*) distance. ${\text{W}}{{\text{V}}_{{\text{bc}}}} = \frac{1}{n}\mathop \sum \limits_{i = 1}^n \frac{{dtw\left( {C,{X_i}} \right)}}{{{l_i}}}$ *C*: medoid beat before clustering, *X_i_: i*th SCG beat, *l_i_*: warping path length, *n* = number of SCG events in a session.	SCG Features
2	Inter-cluster waveform variability (WV_inter_)	Average dissimilarity between the medoid of a cluster and SCG beats of the other cluster. WV_inter_ =$\frac{1}{{n1 + n2}}\left[ {\mathop \sum \limits_{i = 1}^{n1} \frac{{dtw\left( {{C_1},{X_{i2}}} \right)}}{{{l_i}}} + \mathop \sum \limits_{i = 1}^{n2} \frac{{dtw\left( {{C_2},{X_{i1}}} \right)}}{{{l_i}}}} \right]$ *n*1, *n*2: number of events in Cluster 1 and 2, *C*_1_^,^ *C*_2_: SCG medoid of cluster 1 and 2, *X_i_*_1_, *X_i_*_2_: *i*th SCG event of cluster 1 and 2	
3	Intra-cluster waveform variability (WV_intra_)	Average dissimilarity between the medoid and SCG beats of the same cluster WV_intra_ =$\frac{1}{{n1 + n2}}\left[ {\mathop \sum \limits_{i = 1}^{n1} \frac{{dtw\left( {{C_1},{X_{i1}}} \right)}}{{{l_i}}} + \mathop \sum \limits_{i = 1}^{n2} \frac{{dtw\left( {{C_2},{X_{i2}}} \right)}}{{{l_i}}}} \right].$	
4	Average RMS amplitude of instantaneous frequency (*F*_ins_)	Instantaneous frequency (*F*_ins_) was calculated as the frequency first moment of the time–frequency distribution (PCT), normalized by the integral of PCT at that time instant *F*_ins_ =$\frac{{{{\mathop \smallint \nolimits}}_{0.5}^{50}f*{\text{PCT}}\left( {t,f} \right){\text{d}}f}}{{{{\mathop \smallint \nolimits}}_{0.5}^{50}{\text{PCT}}\left( {t,f} \right){\text{d}}f}}.$ Then, the RMS of *F*_ins_ was calculated over the duration of the beats under consideration.	
5	Average turning point ratio (TPR)	${\text{TPR}} = \frac{{N\left( {\left( {{x_i} - {x_{i - 1}}} \right)*\left( {{x_i} - {x_{i + 1}}} \right)} \right) &gt; 0}}{{{\text{length of the signal}}}}.$ Quantification of the randomness in a time-series signal.	

6	Average sample entropy (SmEn)	SmEn = $ - \ln \frac{{{\text{coun}}{{\text{t}}_{m + 1}}\left( {{\text{similar}}} \right)}}{{{\text{coun}}{{\text{t}}_m}\left( {{\text{similar}}} \right)}}$; here denominator and numerator are the number of matched template pairs of length *m* and *m* + 1 in the waveform, respectively (Richman and Moorman 2000).	

7	Average Higuchi dimension (*D*_H_)	Measures the irregularity in a time-series signal (Higuchi 1988).
8	Low frequency power (LFP)	Spectral power of heart rate (HR) in.04–.15 Hz frequency band.	HRV Features
9	High frequency power (HFP)	Spectral power of HR in.15–.4 Hz frequency band.	
10	Total power (TP)	Total spectral power of HR in 0–0.4 Hz frequency band.	
11	pNN50	Proportion of successive RR intervals that differ by more than 50 ms.	

#### 2.2.5. Image construction using time–frequency conversion

**Figure 5.**
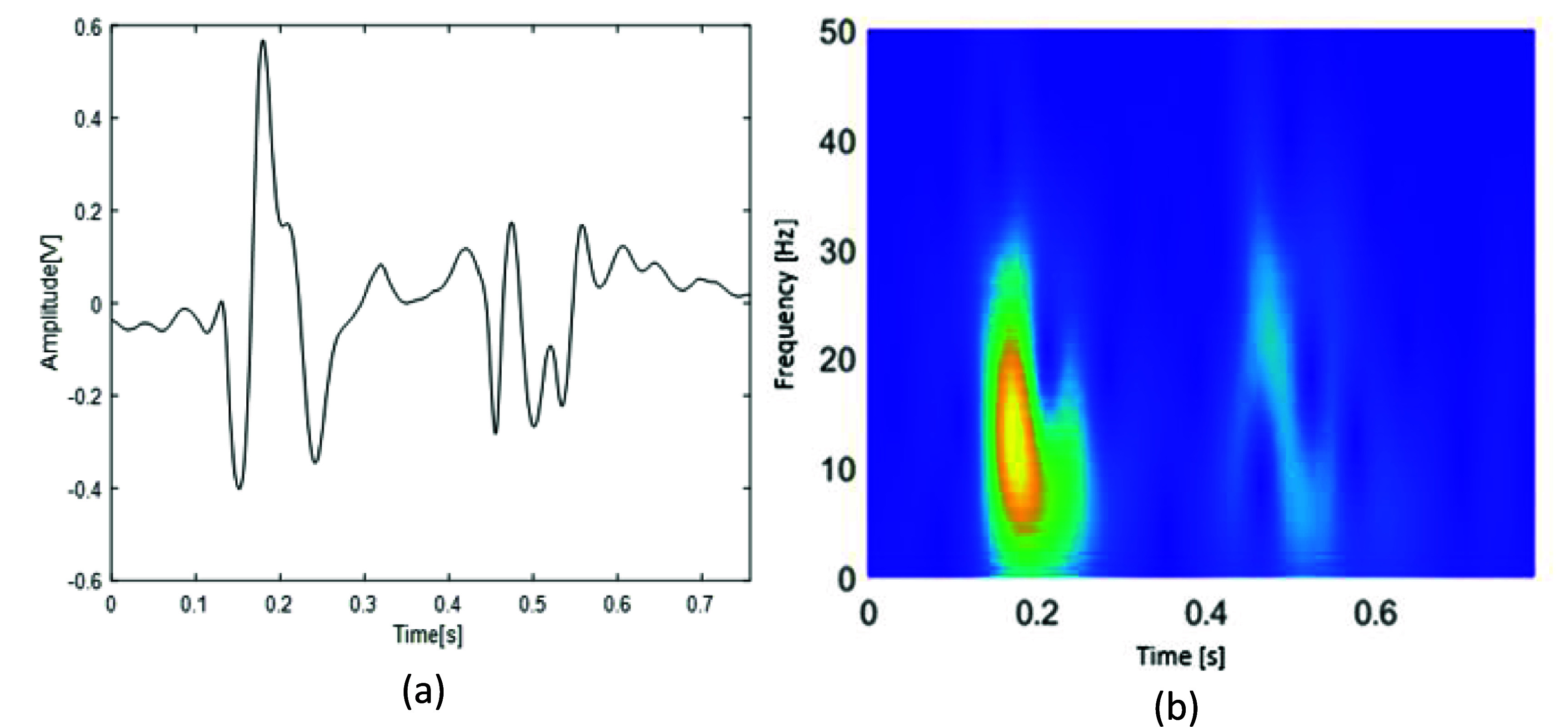
(a) SCG medoid beat of a representative subject (subject 3, session 3), and (b) the corresponding time–frequency distribution coefficient heatmap as calculated by PCT.

For the deep learning approach (approach 2 in figure 2), PCT (a time frequency distribution method) of the SCG signals was calculated and resulted in images. Depending on the length of session data, 3–5 SCG beats closest (as measured by DTW) to the medoid signals were processed by PCT. This resulted in 2D images with time and frequency information in horizontal and vertical axes, respectively (figure 5(b)). The PCT coefficient values were presented using the ‘Parula’ colormap. PCT is found to be more suited than other TFD methods for SCG and heart sound-related studies (Taebi and Mansy 2017, Bao *et al* 2023).

#### 2.2.6. Conventional ML algorithms

Three different ML algorithms were employed to evaluate the efficacy of the feature set in predicting HF readmission. These methods are k-nearest neighbor (KNN), multilayer perceptron neural network (MLP-NN), and extreme gradient boosting (XGBoost). Since there was an imbalance in the number of observations between the two classes, the decision threshold governing the conversion of the prediction probability to a class label was shifted from the default value of 0.5 and tuned to 0.7 to maximize sensitivity. The leave-one-subject-out cross-validation (LOOCV) approach was used for testing to avoid subject bias.

#### 2.2.7. Convolutional neural network

For image classification, the Residual Networks (ResNet-34) model was used. ResNets are being widely used in image classification after being introduced by He *et al* (2015). Several ResNet-based time–frequency image classification tasks have been studied previously (Diker *et al* 2019, Zhang *et al* 2021, Liu *et al* 2022). In this study, a 34-layer CNN network, ResNet-34, was used. Images were resized to 224 by 224 pixels with nearest neighbor interpolation to match the input requirement of ResNet-34. Image augmentation was performed by transformations such as random flips (horizontal and vertical) and rotation. The Adam optimizer with a learning rate of 0.000 008 was chosen. Cross-entropy loss metric was used for performance measurement. The number of epochs was 30 with a batch size of 8.

A balanced dataset, including all the readmitted patients and a subset of non-readmitted patients, was created to address the class imbalance issue for CNN. The number of observations for both the classes was balanced by random undersampling the majority class (non-readmitted patients). This dataset had 38 patients with 90 sessions (22 readmitted with 41 sessions) who were trained and tested by LOOCV. The remaining 43 non-readmitted patients with 52 sessions were not included in the training and only used for out-of-sample testing. These patients were tested using a model trained by data from all the sessions of the 38 patients. This also mimics a real-life application of the developed deep learning model, where the model is trained using the available HF patient data, and the trained model predicts the readmission of the future HF patients.

## 3. Results

Five metrics were used to show the results (equations (1)–(5)),

\begin{equation*}{{\text{sensitivity}}} / {{\text{recall}}} = \frac{{{\text{True positive}}}}{{{\text{True positive} + \text{False negative}}}}\end{equation*}

\begin{equation*}{\text{specificity}} = \frac{{{\text{True negative}}}}{{{\text{True negative} + \text{False positive}}}}\end{equation*}

\begin{equation*}{\text{precision}} = \frac{{{\text{True positive}}}}{{{\text{True positive} + \text{False positive}}}}\end{equation*}

\begin{equation*}F1 - {\text{score}} = \frac{{2*{\text{precision}}*{\text{recall}}}}{{{\text{precision} + \text{recall}}}}\end{equation*}

\begin{equation*}{\text{accuracy}} = \frac{{{\text{True positive} + \text{True negative}}}}{{{\text{True positive} + \text{True negative} + \text{False positive} + \text{False negative }}}}.\end{equation*}

**Figure 6.**
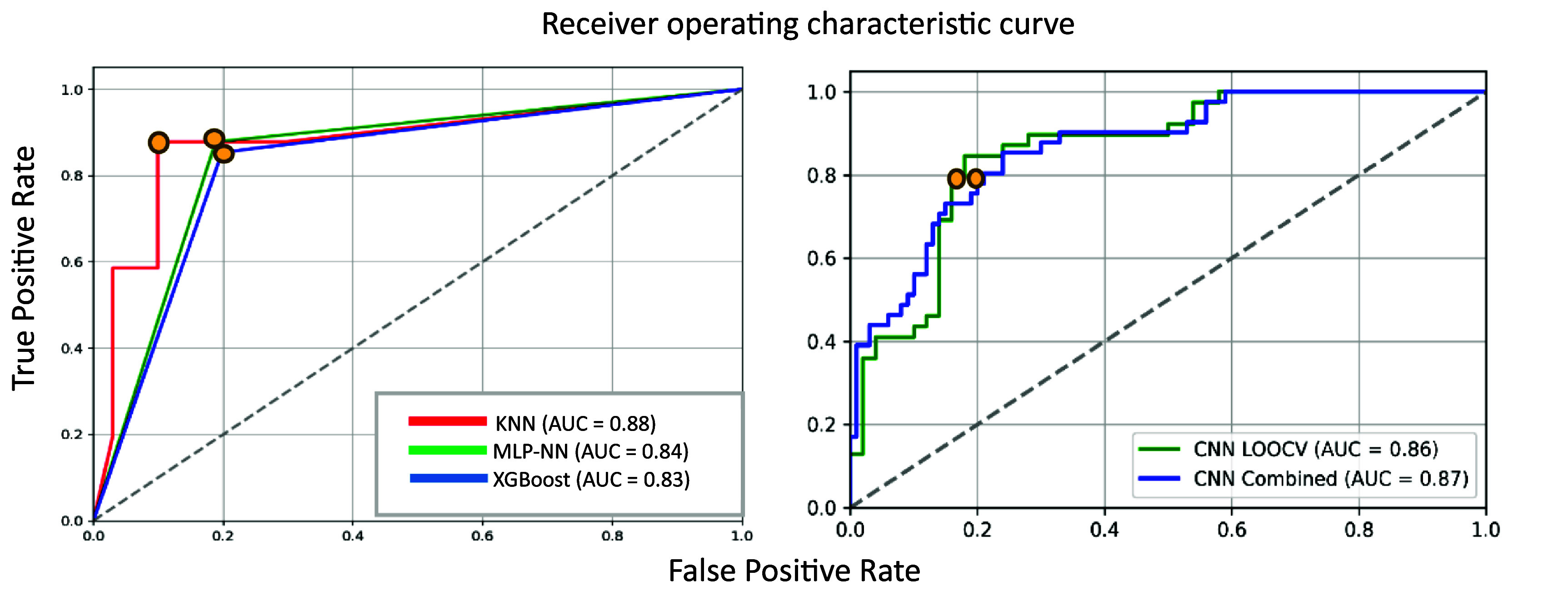
ROC curves for (a) the machine learning models (b) CNN models. The optimum thresholds are indicated by the yellow points (0.7 for ML and 0.5 for CNN).

The results obtained are presented in tables 4 and 5, and the ROC curves are shown in figure 6.

**Table 4.** Performance of the conventional machine learning models, boldface marks the best value in each column.

Model	Sensitivity	Specificity	Precision	*F*1-score	AUC	Accuracy
KNN	**0.88**	**0.90**	**0.78**	**0.83**	**0.88**	**0.89**
MLP-NN	0.88	0.81	0.65	0.75	0.84	0.83
XGBoost	0.85	0.80	0.64	0.73	0.83	0.82

**Table 5.** Performance metrics for the CNN model. 1st row shows the leave-one-subject-out cross-validation (LOOCV) metrics for the balanced dataset (38 patients). The 2nd row shows combined results after adding out-of-sample test set results.

ResNet-34	Sensitivity	Specificity	Precision	*F*1-score	AUC	Accuracy
LOOCV	0.80	0.82	0.78	0.79	0.86	0.81
Combined	0.80	0.81	0.63	0.70	0.87	0.81

These results suggest that conventional ML algorithms performed better than the deep neural network (DNN) model with higher sensitivity. Specifically, KNN outperformed all other models with close to 90% accuracy.

The quantitative comparisons of different HF readmission prediction models are presented in table 6.

**Table 6.** Performance comparison of various methods for predicting patient readmission, boldface marks the best value in each column.

Methods	Subjects (readmitted)	Data type	Accuracy (%)	Sensitivity (%)	Specificity (%)	AUC
Shameer *et al* (2016)	1068 (178)	Electronic medical record	83.19	—	—	0.78
Awan *et al* (2019)	10 757 (2546)	Electronic health record	64.9	48.42	70.01	0.63
Cleland and Antony (2011)	501 (58)	Thoracic impedance	—	42.1		
Stehlik *et al* (2020)	100 (49)	ECG, accelerometry, skin impedance, temperature, activity, posture	—	87.5	86.0	**0.89**
Yu *et al* (2005)	33 (10)	Thoracic impedance	—	76.9		—
Boehmer *et al* (2017)	900 (146)	Heart sounds, thoracic impedance, heart rate, activity, respiration rate	—	70	85.7	—
This study	81 (22)	SCG, ECG, GSR	**88.9**	**87.8**	**90.1**	0.88

## 4. Discussion

A non-invasive approach of predicting HF readmission was proposed and tested in this study. The linear acceleration in dorsal–ventral direction was analyzed and used to classify HF patients (admitted vs non-readmitted). Data analysis was performed in two different approaches: (a) conventional ML and (b) deep learning. In the first approach, features were first extracted from SCG beats and HRV. Feature selection was performed, followed by using three different ML algorithms. For the second approach, time–frequency distribution (PCT) was applied to convert the time-domain signal into a 2D image with time and frequency information. The images were resized and fed into a CNN network (ResNet-34) for classification.

Results showed that handcrafted features provided better accuracy than the CNN method. One reason for this can be the inclusion of HRV features in the feature set, which was not provided to the CNN model. Given the higher performance of conventional ML models (with the SCG and HRV features), a discussion of these features that correlate those with HF conditions may be useful. The focus here will be given to SCG clustering features and HRV features.

**Figure 7.**
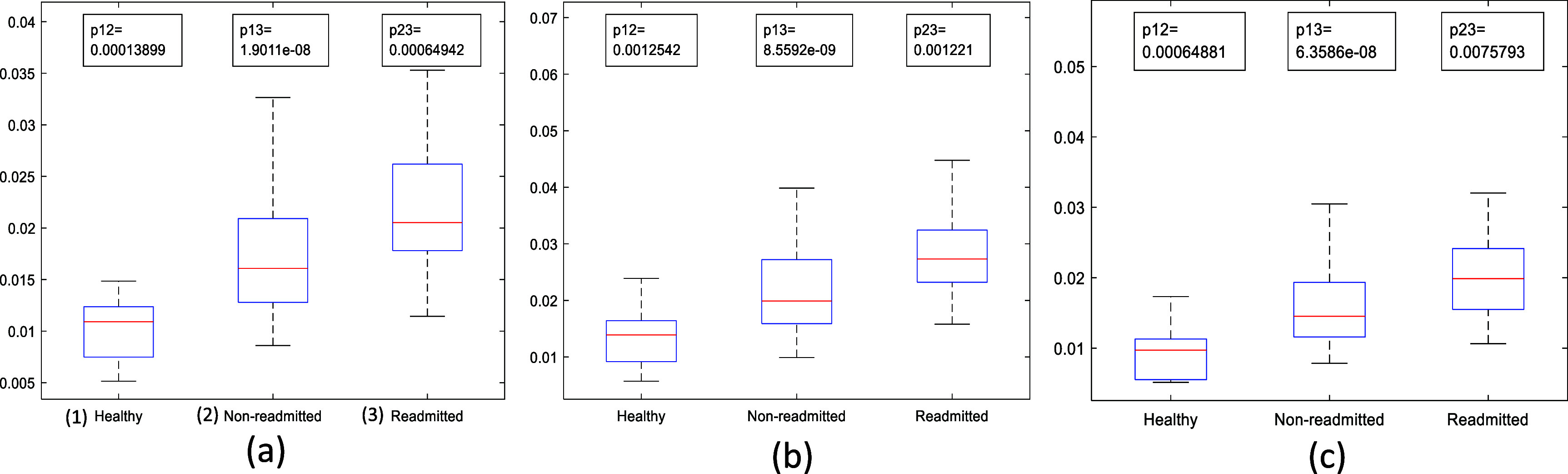
Shows the feature values of (1) healthy, (2) non-readmitted, and (3) readmitted groups in a boxplot for (a) intra-session waveform variability before clustering, (b) inter-cluster variability, and (c) intra-cluster variability features. All the feature values are highest in the readmitted patient group and lowest in the healthy group. The differences between each pair of the groups are statistically significant as depicted by p-values (two-sample t-test) at the top of each image. Here, the numbers beside ‘p’ indicate the numbers of the groups being compared (1-healthy, 2-non-readmitted groups, 3-readmitted).

The first three features in table 3 are the SCG clustering features. The first feature is intra-session waveform variability calculated before clustering. This feature represents the dissimilarity among SCG beats during a session. Inter- and intra-cluster variability features were also obtained after clustering. These features present the average dissimilarity of SCG beats between and within the clusters, respectively. Overall, these clustering features indicate the beat-to-beat waveform variability. The distributions of the clustering feature values in non-readmitted and readmitted patient groups are shown in figure 7. For comparison, feature values of a group of 14 healthy subjects are also shown. Data was acquired from the healthy subjects using the same protocol.

HF is associated with chronic sympathetic/parasympathetic imbalance resulting in increased sympathetic and decreased parasympathetic drive (Binkley *et al* 1991, Mann 1999, Braunwald and Bristow 2000, Floras 2009). This also decreases peripheral acetylcholine (ACh) secretion (Roy *et al* 2014). ACh is the main neurotransmitter of the parasympathetic nervous system (Sam and Bordoni 2023). Binding inhibition of ACh to receptors in the heart has several effects, such as increasing heart rate and heart contraction force, etc (Galper and Smith 1978, Moss *et al* 2018). In fact, increasing ACh might be a logical HF treatment since it may reverse the effect of decreased ACh with HF (Roy *et al* 2014, Koncz *et al* 2022).

The trend of increased beat-to-beat SCG waveform variability with worsened HF (see figure 7) may be explained by the decreased acetylcholine (ACh) release in HF. In an animal study, Ahammer *et al* reported that decreased ACh increased beat-to-beat contraction strength variability of murine atrial preparation (2018). In that study, hearts were removed, and the atria were dissected from the ventricles. Variability analysis of contraction strengths was performed under control and ACh-treated conditions. Variability of contraction strength was significantly higher in control tissue (which had lower ACh). This suggests that decreased ACh in HF may play a role in increasing the beat-to-beat variability of cardiac contraction. Increased cardiac contraction variability (associated with decreased ACh secretion) is believed to be a major contributor to SCG signal variability (Rienzo *et al* 2013, Taebi *et al* 2019). The lowest variability was found in the healthy group (figure 7), which further strengthens this argument.

**Figure 8.**
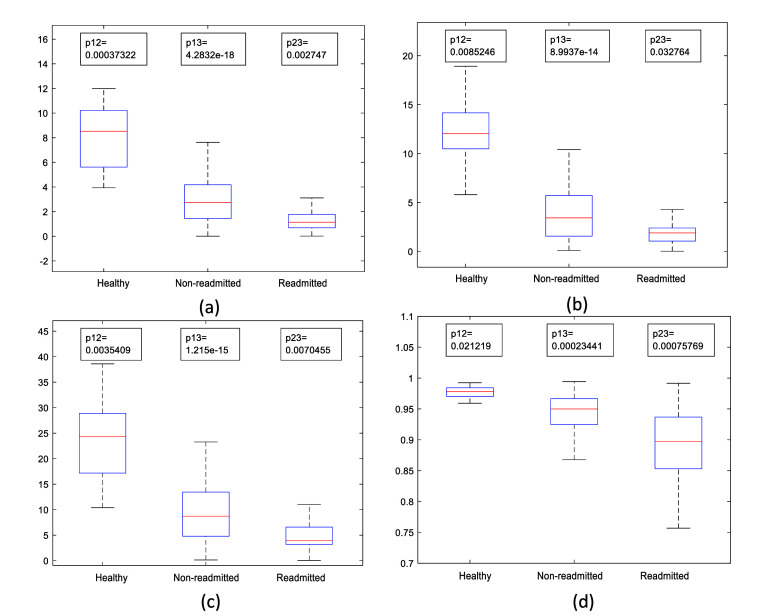
Shows the feature values of (1) healthy, (2) non-readmitted and, (3) readmitted groups in boxplot for (a) LFP, (b) HFP, (c) TP and (d) pNN50. HRV feature values are highest in healthy group and lowest in the readmitted patient group. P-values obtained by two sample t-test demonstrate that each pair of the groups are significantly different.

Another important factor to be considered here is the trend in HRV features. Figure 8 shows the boxplots of selected HRV features for the 3 groups of subjects.

It is evident from figure 8 that, compared to healthy subjects, HRV features decline in non-readmitted HF patients, then decline further in readmitted HF patients. This can also be due to cardiovascular autonomic imbalance, as HRV is increased by parasympathetic nervous activation and decreased by sympathetic nervous system activation (Berntson *et al* 1997).

The following are some of the important takeaways from this research:

1. The current study demonstrates the potential use of SCG signals in HF readmission prediction.
2. In this study, conventional ML algorithms, especially KNN, outperformed the DNN model. Other than adding the HRV features, an extensive dataset would benefit the DNN model. In the future, the performance of lightweight DNN models should be evaluated and compared with current study results.
3. Future analysis should include SCG signal in two other directions (lateral and caudocranial axes). The inclusion of a 3-axis gyroscope sensor in the protocol would cover more complete cardiac movement by incorporating angular velocity of the heart. This can elicit more useful features related to HF readmission. Additionally, adding ERHs can improve the classification performance of the models.
4. The advantage of using handcrafted features is the interpretability of the features. Extracting features based on physiological knowledge can make the results more meaningful and reveal underlying characteristics of the data. On the other hand, the use of the DNN model eliminated the need for manual feature engineering at the cost of interpretability. The future work of this study would be to focus on understanding the DNN model results by incorporating explainable AI techniques.
5. More patient data is required to confirm the current study results and apply them in clinical settings.
6. The possibility that noncardiac comorbidities such as chronic kidney disease, diabetes, dementia, etc could be the cause of an HF readmission is one study limitation. SCG is limited to predicting the readmissions associated with cardiac conditions.
7. More gender-balanced data should be collected to compare the outcomes for the two sexes. Age matching between healthy subjects and patients could not be achieved due to the unavailability of age information for the patient group. In future studies, efforts should be made to record and incorporate age data to enable more accurate comparisons.

## 5. Conclusion

This study describes a non-invasive technique to predict HF readmission. SCG, ECG, and GSR signals were acquired from non-readmitted and readmitted HF patients as well as normal subjects. After preprocessing and feature extraction, conventional ML algorithms and deep learning models were applied to classify the two patient groups. Results showed that the KNN model achieved the highest classification accuracy of about 90%. This suggests that SCG signal has potential utility for monitoring patients with cardiac disease. Early HF readmission prediction may potentially help the clinicians to identify the patients who need special care and treatment and make rapid targeted interventions to avoid readmission. This will ensure better management of HF patients and reduce the mortality rate. More patient populations with different cardiac conditions may be added for clinical application of SCG signals in the future.

## Data Availability

The data cannot be made publicly available upon publication due to legal restrictions preventing unrestricted public distribution. The data that support the findings of this study are available upon reasonable request from the authors.

## References

[pmeaae178cbib1] Ahammer H, Scheruebel S, Arnold R, Mayrhofer-Reinhartshuber M, Lang P, Dolgos Á, Pelzmann B, Zorn-Pauly K (2018). Sinoatrial beat to beat variability assessed by contraction strength in addition to the interbeat interval. Front. Physiol..

[pmeaae178cbib2] Awan S E, Bennamoun M, Sohel F, Sanfilippo F M, Dwivedi G (2019). Machine learning-based prediction of heart failure readmission or death: implications of choosing the right model and the right metrics. ESC Heart Fail..

[pmeaae178cbib3] Azad K, Gamage P T, Sandler R H, Raval N, Mansy H A (2018). Detection of respiratory phase and rate from chest surface measurements. J. Appl. Biotechnol. Bioeng..

[pmeaae178cbib4] Azad M K, Gamage P T, Dhar R, Sandler R H, Mansy H A (2023). Postural and longitudinal variability in seismocardiographic signals. Physiol. Meas..

[pmeaae178cbib5] Azad M K, Gamage P T, Sandler R H, Raval N, Mansy H A (2019). Seismocardiographic signal variability during regular breathing and breath hold in healthy adults.

[pmeaae178cbib6] Bao X, Xu Y, Lam H-K, Trabelsi M, Chihi I, Sidhom L, Kamavuako E N (2023). Time-frequency distributions of heart sound signals: a Comparative study using convolutional neural networks. Biomed. Eng. Adv..

[pmeaae178cbib7] Berntson G G (1997). Heart rate variability: origins, methods, and interpretive caveats. Psychophysiology.

[pmeaae178cbib8] Binkley P F, Nunziata E, Haas G J, Nelson S D, Cody R J (1991). Parasympathetic withdrawal is an integral component of autonomic imbalance in congestive heart failure: demonstration in human subjects and verification in a paced canine model of ventricular failure. J. Am. College Cardiol..

[pmeaae178cbib9] Boehmer J P (2017). A multisensor algorithm predicts heart failure events in patients with implanted devices results from the MultiSENSE study.

[pmeaae178cbib10] Bozkurt B (2023). Mortality, outcomes, costs, and use of medicines following a first heart failure hospitalization. JACC Heart Fail..

[pmeaae178cbib11] Braunwald E, Bristow M R (2000). Congestive heart failure: fifty years of progress. Circulation.

[pmeaae178cbib12] Burkhoff D (2012). Mortality in heart failure with preserved ejection fraction: an unacceptably high rate. Eur. Heart J..

[pmeaae178cbib13] Cleland J G F, Antony R (2011). It makes SENSE to take a safer road. Eur. Heart J..

[pmeaae178cbib14] Cui X, Thunström E, Dahlström U, Zhou J, Ge J, Fu M (2020). Trends in cause-specific readmissions in heart failure with preserved vs. reduced and mid-range ejection fraction. ESC Heart Fail..

[pmeaae178cbib15] Desai A S, Stevenson L W (2012). Rehospitalization for heart failure: predict or prevent?. Circulation.

[pmeaae178cbib16] Diker A, Comert Z, Avci E, Togacar M, Ergen B (2019). A novel application based on spectrogram and convolutional neural network for ECG classification.

[pmeaae178cbib17] Floras J S (2009). Sympathetic nervous system activation in human heart failure: clinical implications of an updated model. J. Am. College Cardiol..

[pmeaae178cbib18] Galper J B, Smith T W (1978). Properties of muscarinic acetylcholine receptors in heart cell cultures. Proc. Natl Acad. Sci..

[pmeaae178cbib19] Gamage P T, Azad M K, Taebi A, Sandler R H, Mansy H A (2020). Clustering of SCG events using unsupervised machine learning. Signal Processing in Medicine and Biology: Emerging Trends in Research and Applications.

[pmeaae178cbib20] Guo Y, Chung F-L, Li G, Zhang L (2019). Multi-label bioinformatics data classification with ensemble embedded feature selection. IEEE Access.

[pmeaae178cbib21] He K, Zhang X, Ren S, Sun J (2015). Deep residual learning for image recognition. https://arxiv.org/abs/1512.03385.

[pmeaae178cbib22] Heidenreich P A (2022). 2022 AHA/ACC/HFSA guideline for the management of heart failure: a report of the American college of cardiology/American heart association joint committee on clinical practice guidelines. Circulation.

[pmeaae178cbib23] Heist E K, Herre J M, Binkley P F, Bakel A B V, Porterfield J G, Porterfield L M, Qu F, Turkel M, Pavri B B (2014). Analysis of different device-based intrathoracic impedance vectors for detection of heart failure events (from the detect fluid early from intrathoracic impedance monitoring study). Am. J. Cardiol..

[pmeaae178cbib24] Higuchi T (1988). Approach to an irregular time series on the basis of the fractal theory. Physica D.

[pmeaae178cbib25] Inan O T (2018). Novel wearable seismocardiography and machine learning algorithms can assess clinical status of heart failure patients. Circulation.

[pmeaae178cbib26] Jones N R, Roalfe A K, Adoki I, Hobbs F D R, Taylor C J (2019). Survival of patients with chronic heart failure in the community: a systematic review and meta-analysis. Eur. J. Heart Fail..

[pmeaae178cbib27] Khan M S, Sreenivasan J, Lateef N, Abougergi M S, Greene S J, Ahmad T, Anker S D, Fonarow G C, Butler J (2021). Trends in 30- and 90-Day readmission rates for heart failure. Circulation.

[pmeaae178cbib28] Koncz I (2022). Acetylcholine reduces IKr and prolongs action potentials in human ventricular cardiomyocytes. Biomedicines.

[pmeaae178cbib29] Lang R M (2015). Recommendations for cardiac chamber quantification by echocardiography in adults: an update from the American society of echocardiography and the European Association of cardiovascular imaging. J. Am. Soc. Echocardiogr..

[pmeaae178cbib30] Lesyuk W, Kriza C, Kolominsky-Rabas P (2018). Cost-of-illness studies in heart failure: a systematic review 2004-2016. BMC Cardiovasc. Disorders.

[pmeaae178cbib31] Lin W-Y, Ke H-L, Chou W-C, Chang P-C, Tsai T-H, Lee M-Y (2018). Realization and technology acceptance test of a wearable cardiac health monitoring and early warning system with multi-channel MCGs and ECG. Sensors.

[pmeaae178cbib32] Liu J, Liu P, Lei M-R, Zhang H-W, You A-L, Luan X-R (2022). Readmission risk prediction model for patients with chronic heart failure: a systematic review and meta-analysis. Iran. J. Public Health.

[pmeaae178cbib33] Mann D L (1999). Mechanisms and models in heart failure: a combinatorial approach. Circulation.

[pmeaae178cbib34] Moss R, Sachse F B, Moreno-Galindo E G, Navarro-Polanco R A, Tristani-Firouzi M, Seemann G, Marsden A L (2018). Modeling effects of voltage dependent properties of the cardiac muscarinic receptor on human sinus node function. PLOS Comput. Biol..

[pmeaae178cbib35] Murphy S P, Ibrahim N E, Januzzi J L (2020). Heart failure with reduced ejection fraction: a review. JAMA.

[pmeaae178cbib36] Pudjihartono N, Fadason T, Kempa-Liehr A W, O’Sullivan J M (2022). A review of feature selection methods for machine learning-based disease risk prediction. Front. Bioinform..

[pmeaae178cbib37] Richman J S, Moorman J R (2000). Physiological time-series analysis using approximate entropy and sample entropy. Am. J. Physiol. Heart. Circ. Physiol..

[pmeaae178cbib38] Rienzo M D, Vaini E, Castiglioni P, Merati G, Meriggi P, Parati G, Faini A, Rizzo F (2013). Wearable seismocardiography: towards a beat-by-beat assessment of cardiac mechanics in ambulant subjects. Auton Neurosci..

[pmeaae178cbib39] Roy A, Guatimosim S, Prado V F, Gros R, Prado M A M (2014). Cholinergic activity as a new target in diseases of the heart. Mol. Med..

[pmeaae178cbib40] Sam C, Bordoni B (2023). Physiology, acetylcholine. StatPearls.

[pmeaae178cbib41] Sandler R H, Azad K, Rahman B, Taebi A, Gamage P, Raval N, Mentz R J, Mansy H A (2019). Minimizing seismocardiography variability by accounting for respiratory effects. J. Card Fail.

[pmeaae178cbib42] Savarese G, Lund L H (2017). Global public health burden of heart failure. Cardiac. Fail. Rev..

[pmeaae178cbib43] Shafie A A, Tan Y P, Ng C H (2018). Systematic review of economic burden of heart failure. Heart Failure Rev..

[pmeaae178cbib44] Shameer K (2016). Predictive modeling of hospital readmission rates using electronic medical record-wide machine learning: a case-study using Mount Sinai heart failure cohort Sinai Health System at Mount Sinai 3 Decision Support, Mount Sinai Health System at Mount Sinai 4 Mount Sinai Data Warehouse, Icahn Institute of Genomics and Multiscale Biology at Mount Sinai HHS Public Access. Pac. Symp. Biocomput..

[pmeaae178cbib45] Sheikh W, Bilal Ahmed M, Parulkar A, Lhungay T, Sharma E, Kennedy K, Ahmed Z, Lima F, Aronow H, Chu A (2021). Association between the hospital readmissions reduction program and heart failure subtype readmissions and mortality in the USA. EMJ Cardiol..

[pmeaae178cbib46] Somaratne J B, Berry C, McMurray J J V, Poppe K K, Doughty R N, Whalley G A (2009). The prognostic significance of heart failure with preserved left ventricular ejection fraction: a literature-based meta-analysis. Eur. J. Heart Fail..

[pmeaae178cbib47] Stauffer B D (2011). Effectiveness and cost of a transitional care program for heart failure: a prospective study with concurrent controls. Arch. Int. Med..

[pmeaae178cbib48] Stehlik J (2020). Continuous wearable monitoring analytics predict heart failure hospitalization: the LINK-HF multicenter study. Circulation.

[pmeaae178cbib49] Taebi A, Mansy H A (2017). Time-frequency distribution of seismocardiographic signals: a comparative study. Bioengineering.

[pmeaae178cbib50] Taebi A, Solar B, Bomar A, Sandler R, Mansy H (2019). Recent advances in seismocardiography. Vibration.

[pmeaae178cbib51] Tompkins W J (1985). A real-time QRS detection algorithm. IEEE Trans. Biomed. Eng..

[pmeaae178cbib52] Urbich M, Globe G, Pantiri K, Heisen M, Bennison C, Wirtz H S, Tanna G L D (2020). A systematic review of medical costs associated with heart failure in the USA (2014–2020). PharmacoEconomics.

[pmeaae178cbib53] Virani S S (2020). Heart disease and stroke statistics—2020 update a report from the American heart association. Circulation.

[pmeaae178cbib54] Yu C-M, Wang L, Chau E, Chan R H-W, Kong S-L, Tang M-O, Christensen J, Stadler R W, Lau C-P (2005). Intrathoracic impedance monitoring in patients with heart failure: correlation with fluid status and feasibility of early warning preceding hospitalization. Circulation.

[pmeaae178cbib55] Zhang Y, Li J, Wei S, Zhou F, Li D (2021). Heartbeats classification using hybrid time-frequency analysis and transfer learning based on ResNet. IEEE J. Biomed. Health Inform..

